# Supplementary material for: Analyzing the Quantum Zeno and anti-Zeno effects using optimal projective measurements
Source: Sci Rep. 2017 Sep 18;7:11766. doi: 10.1038/s41598-017-11787-9 (PMC5603598; doi:10.1038/s41598-017-11787-9)
Supplement: Supplementary file 1 — Supplementary Information [file 41598_2017_11787_MOESM1_ESM.pdf]

Supplemental Material: Analyzing the Quantum Zeno and anti-Zeno effects using optimal projective measurements

Muhammad Junaid Aftab[1] and Adam Zaman Chaudhry[1,*]

[1]*School of Science & Engineering, Lahore University of Management Sciences (LUMS), Opposite Sector U, D.H.A, Lahore 54792, Pakistan*

In the first part of the Supplementary Material, we look at what happens to the optimized effective decay rate if we do not have perfect information about the system-environment parameters. In the second part, we look briefly at the effect of non-ideal measurements.

I. ERRORS IN THE SYSTEM-ENVIRONMENT PARAMETERS

In the main text, we derived the expression of the optimal survival probability assuming one makes repeated optimal projective measurements. This expression depends on $\mathbf{n}(\tau)$, the Bloch vector of the density matrix of the system at time τ. It should be kept in mind that our treatment assumed that the evolution of the density matrix of the system is known perfectly. However, a complete knowledge of the evolution of the density matrix of the system depends on knowing perfectly a number of factors such as the parameters in the system Hamiltonian, the parameters characterizing the environment with which the system has been coupled to and the form of the coupling term between the system and the environment. In reality, we will not have such complete information. To analyze the effect of not having such complete information, we assume that there is some error in our knowledge of one of these parameters. That is, consider that the value of a particular system-environment parameter, say κ, is equal to some value, c. We, however, are under the assumption that the value of the parameter is c'. The optimal projective measurement that we will choose to perform will be derived on the basis of the system-environment parameter being equal to c'. However, the system density matrix actually evolves according the system-environment parameter being equal to c. In this case, the expression for the survival probability is,

$$s(\tau) = \frac{1}{2}\left(1 + \mathbf{n}_c(\tau)\cdot\mathbf{n}_{c'}(\tau)\right) = \frac{1}{2}\left(1 + \|\mathbf{n}_\mathbf{c}(\tau)\|\cdot\|\mathbf{n}_{\mathbf{c}'}(\tau)\|\cos\theta\right), \tag{1}$$

where $\mathbf{n}_c(\tau)$ is the value of the Bloch vector of the density matrix of the system when $\kappa = c$, and $\mathbf{n}_{c'}(\tau)$ is the value of the Bloch vector of the density matrix of the system when $\kappa = c'$. We expect equation (1) to yield a smaller probability compared to the expression of the survival probability due to the additional factor of $\cos\theta$, where θ is the angle between the two Bloch vectors.

We now consider the effect of errors in different system-environment parameters one by one. Our objective is to demonstrate that the decay rates obtained with errors are quite close to the optimal decay rates at relatively long times, even if the errors are very significant. It should be kept in mind that, as emphasized in the main text, it is only at relatively long times that our strategy pays dividends in increasing the survival probability of the quantum state as compared to the usual strategy of preparing the same initial quantum state repeatedly.

A. Error in the system-environment coupling strength G

We first consider noise in the value of the the parameter G, the coupling strength between the system and the environment. We calculate the effective decay rate considering no errors (which we call the 'optimal decay rate'), and we also perform the calculation assuming an error in our value of G (which we call the 'unoptimal decay rate'). The results as presented for both the pure dephasing and the more general spin-boson models in Figs. 1 and 2 respectively. A few comments are in order. First, as expected, for small measurement intervals, there is barely any difference between the optimal and unoptimal cases simply because the quantum state does not have enough time to evolve. Second, more importantly, even though the errors can be very large (for instance, in Fig. 1 we consider 100% and 400% errors), at relatively long times, the error has little effect. Since the optimal decay rate has already been shown to be considerably better than the usual decay rate (obtained by measuring the same initial quantum state

*Electronic address: adam.zaman@lums.edu.pk

repeatedly) at long measurement intervals in the main text, and the errors have little effect at long measurement intervals, we can rest assured that our strategy to decrease the effective decay rate is quite robust against errors in the system-environment coupling strength. For more quantitative comparisons, Figs. 1 and Figs. 2 should be compared with Fig. 2(b) and 4(b) of the main text where the optimal decay rate is compared with the usual decay rate.

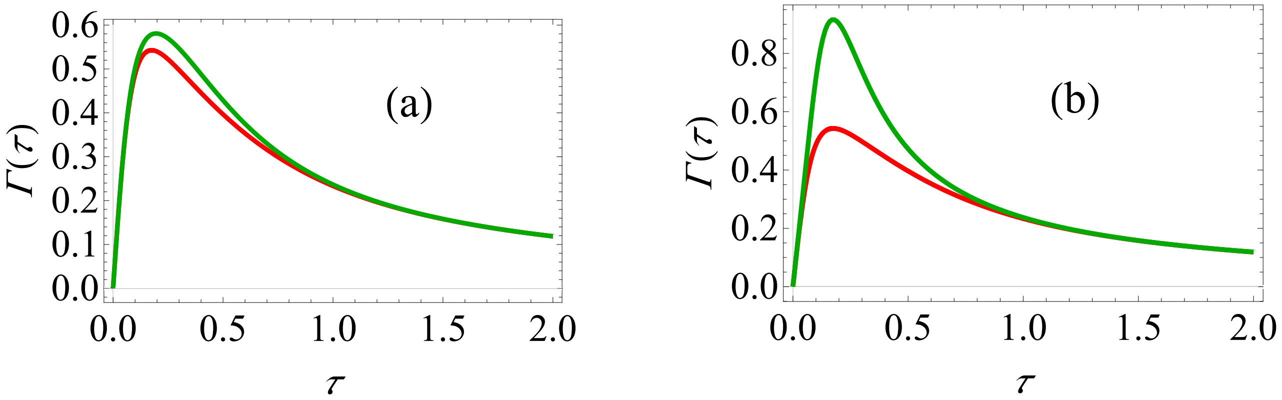

FIG. 1: **Graphs of the optimal and the unoptimal effective decay rate under noise in the value of G in the pure dephasing model.** **(a)** $\Gamma(\tau)$ versus τ for the initial state specified by the Bloch vector $(1/\sqrt{3}, 1/\sqrt{3}, 1/\sqrt{3})$. The red curve shows the optimal decay rate when $G = 0.1$. The green curve shows the unoptimal decay rate when $G' = 0.2$. **(b)** Same as in **(a)** except that $G' = 0.5$. The values of the other parameters used are $\beta = 0.5$ and $\omega_c = 10$.

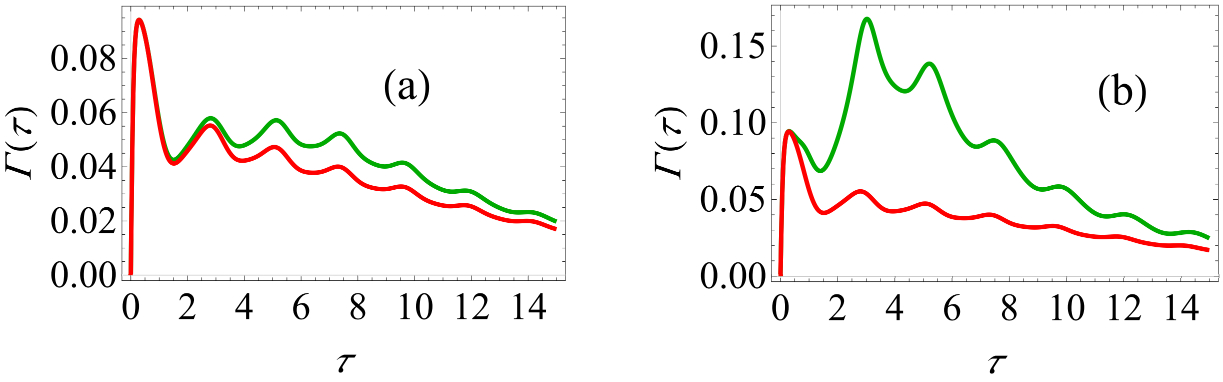

FIG. 2: **Graphs of the optimal and the unoptimal effective decay rate under noise in the value of G in the spin boson model.** **(a)** $\Gamma(\tau)$ versus τ for the initial state specified by $\theta = \pi/2$ and $\alpha = 0$. The red curve shows the optimal decay rate when $G = 0.01$. The green curve shows the unoptimal decay rate when $G' = 0.0175$. **(b)** Same as in **(a)** except that $G' = 0.04$. The values of the other parameters used are $\varepsilon = 2, \Delta = 2, \beta = 1000$, $\omega_c = 10$, and $s = 1$.

B. Error in ω_c

We now carry out a very similar exercise, except that we now consider an error in the value of the cutoff frequency of the environment ω_c. We again consider both the pure dephasing and the spin-boson models. The results are presented in Fig. 3. Once again, our strategy is very robust against errors.

C. Error in the system Hamiltonian parameter ε

We now consider an error in the value of the the system energy gap ε, which is a parameter in the system Hamiltonian in the pure dephasing model. The results shown in Fig. 4 again show that our scheme is robust against errors in ε as well.

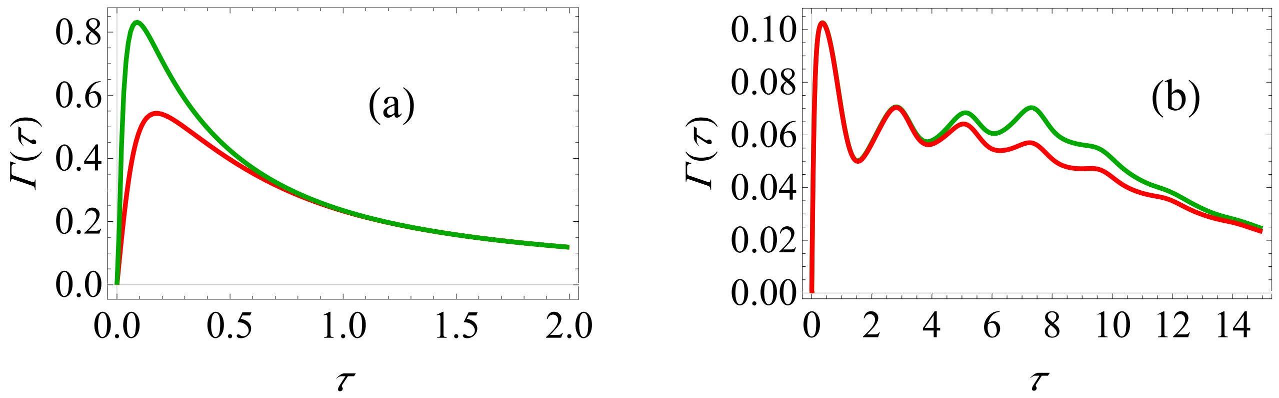

FIG. 3: **Graphs of the optimal and the unoptimal effective decay rate under error in the value of ω_c in the pure dephasing model and the spin boson model.** **(a)** $\Gamma(\tau)$ versus τ for the initial state specified by the Bloch vector $(1/\sqrt{3}, 1/\sqrt{3}, 1/\sqrt{3})$ in the pure dephasing model. The red curve shows the optimal decay rate when $\omega_c = 10$. The green curve shows the unoptimal decay rate when $\omega_c' = 100$. The values of the other parameters used are $\beta = 0.5$ and $G = 0.1$. **(b)** $\Gamma(\tau)$ versus τ for the initial state specified by $\theta = \pi/2$ and $\alpha = 0$ in the spin boson model. Here, we have set $\omega_c' = 50$. The values of the other parameters used are $\varepsilon = 2, \Delta = 2, \beta = 1$, $G = 0.01$, and $s = 1$.

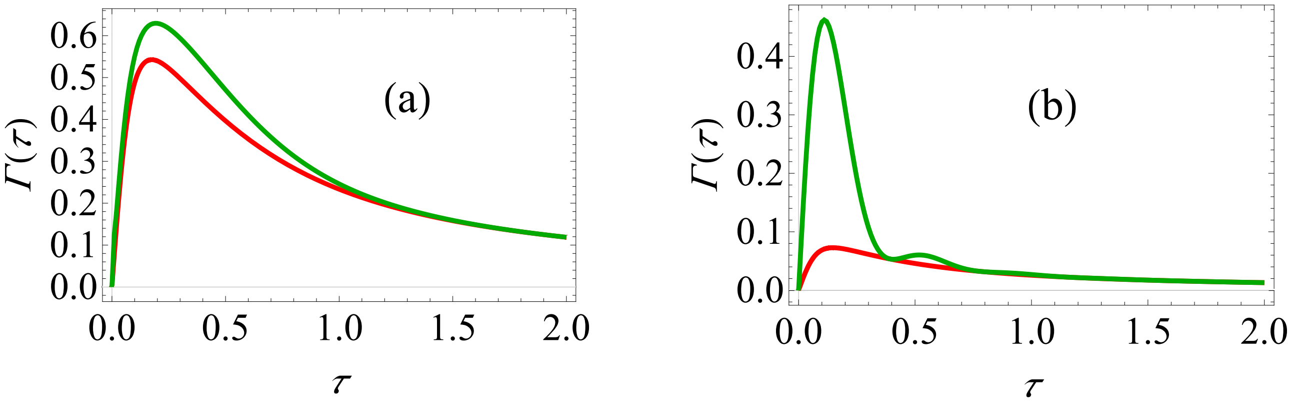

FIG. 4: **Graphs of the optimal and the unoptimal effective decay rate under error in the value of ε in the pure dephasing model.** **(a)** $\Gamma(\tau)$ versus τ for the initial state specified by the Bloch vector $(1/\sqrt{3}, 1/\sqrt{3}, 1/\sqrt{3})$. The red curve shows the optimal decay rate when $\varepsilon = 2$. The green curve shows the unoptimal decay rate when $\varepsilon' = 4$. **(b)** Same as in **(a)** except that now we used the state specified by the Bloch vector $(1/\sqrt{10}, 0, \sqrt{9/10})$, and the value of $\varepsilon' = 18$. The values of the other parameters used are $\beta = 0.5$ and $\omega_c = 10$.

D. Error in the system-environment coupling

Finally, we consider the case where the system is coupled differently to the environment compared to what we anticipate. To model this situation, we consider the spin-boson model. The system and the environment are coupled via the operator $F = \sigma_z$, but we think that they are actually coupled via the operator $F' = \sigma_z + 0.2\sigma_x$. We can again compute the corresponding Bloch vectors and thereafter the optimal and unoptimal effective decay rate. Results are shown in Fig. 5. Once again, our scheme is robust.

II. NON-IDEAL MEASUREMENTS

In this section, we consider the deviation from the optimal effective decay rate if a non-ideal measurement is made. Let us recall that in our scheme, the density matrix of the system at time τ (with the system's unitary evolution removed) is given by

$$\tilde{\rho}_S(\tau) = U_S^\dagger(\tau)\rho_S(\tau)U_S(\tau) \tag{2}$$

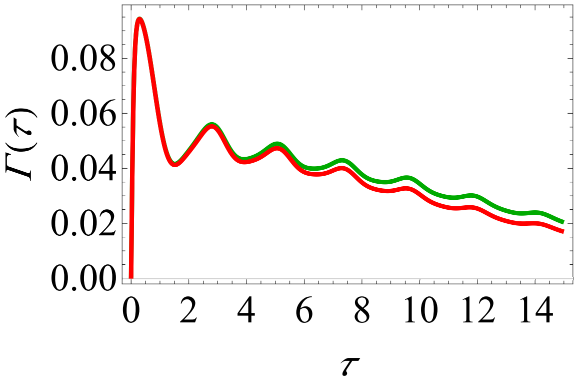

FIG. 5: **Graph of the optimal and the unoptimal effective decay rate under noise in the functional form of F in the spin boson model.** $\Gamma(\tau)$ versus τ for the initial state specified by $\theta = \pi/2$ and $\alpha =$ in the spin-boson model. The red curve shows the optimal decay rate when $F = \sigma_z$. The green curve shows the unoptimal decay rate with $F' = \sigma_z + 0.2\sigma_x$. The values of the other parameters used are $\varepsilon = 2, \Delta = 2, \beta = 1000$, $\omega_c = 10$, $G = 0.01$, and $s = 1$. The inset traces the value of θ/π (the normalized angle between the Bloch vectors).

The unitary operators are there to remove the evolution due to the system evolution itself. We thereafter apply the projector $|\chi\rangle\langle\chi|$, and the corresponding survival probability is given by the expression,

$$s(\tau) = \mathrm{Tr}\big(|\chi\rangle\langle\chi|\,\tilde{\rho}_S(\tau)\big) \tag{3}$$

For the case of a two level system, we know the optimal projector $|\chi\rangle\langle\chi|$. To actually implement this projector, we instead implement the unitary operator U_R such that

$$|\chi\rangle = U_R\,|e\rangle\,, \tag{4}$$

and consequently implement the projector $|e\rangle\langle e|$. This measurement process will not be perfect since there will be errors in implementing the unitary operator U_R as well as the projector $|e\rangle\langle e|$. We will now model these errors. To simplify our analysis, we consider the pure dephasing model with the initial state such that $n_y(0) = 0$. Then,

$$|\chi\rangle = \exp\left(\frac{-i\theta_0(\tau)\sigma_y}{2}\right)|e\rangle\,,$$

Here,

$$\theta_0(\tau) = \arccos\left(\frac{n_z(\tau)}{\sqrt{n_x(\tau)^2 + n_y(\tau)^2 + n_z(\tau)^2}}\right).$$

To model the fact that U_R is now not perfectly implemented, we assume that the angle in the exponent for U_R is distributed normally about $\theta_0(\tau)$ for each value of τ. Furthermore, the measurement fidelity when the projector $|e\rangle\langle e|$ is implemented is denoted by p_e which need not be one. Then, $U_R\,|e\rangle\langle e|\,U_R^\dagger$ becomes

$$\Pi = p_e \int_{-\infty}^{\infty} d\theta\,\underbrace{\frac{1}{\sqrt{2\pi}\sigma}e^{-\frac{(\theta-\theta_0)^2}{2\sigma^2}}}_{G(\theta)}\,e^{-i\theta\sigma_y/2}\,|e\rangle\langle e|\,e^{i\theta\sigma_y/2},$$

and σ is assumed to be a small fraction of θ_0. Simplifying, we obtain

$$\Pi = p_e\Big[I_1\,|e\rangle\langle e| + I_2\,|g\rangle\langle g| + I_3\,|e\rangle\langle g| + I_3\,|g\rangle\langle e|\Big], \tag{5}$$

where

$$I_1 = \int_{-\infty}^{\infty} d\theta\; G(\theta)\cos^2(\theta/2)$$

$$I_2 = \int_{-\infty}^{\infty} d\theta\; G(\theta)\sin^2(\theta/2) \equiv 1 - I_1$$

$$I_3 = \int_{-\infty}^{\infty} d\theta\; G(\theta)\cos(\theta/2)\sin(\theta/2) = \frac{1}{2}\int_{-\infty}^{\infty} d\theta\; G(\theta)\sin(\theta)$$

These integrals can be evaluated. We find that

$$I_1 = \frac{1}{2}(e^{-\sigma^2/2}\cos\theta_0 + 1),$$

which at once implies that,

$$I_2 \equiv 1 - I_1 = \frac{1}{2}(1 - e^{-\sigma^2/2}\cos\theta_0).$$

Similarly,

$$I_3 = \frac{e^{-\sigma^2/2}\sin(\theta_0)}{2}.$$

We can now compute the survival probability

$$s(\tau) = \mathrm{Tr}\big[\Pi\tilde{\rho}_S(\tau)\big],$$

from which the effective decay rate is computed using

$$\Gamma(\tau) = -\frac{1}{\tau}\ln s(\tau).$$

We now present the results for the initial state $n_x(0) = \sqrt{1/10}$ and $n_z = \sqrt{9/10}$ [see Fig. 6]. First, we observe that the decay rate is extremely sensitive to the value of the measurement fidelity p_e. A very high value of p_e ensures that the decay rate under making non ideal measurements (green curve) is approximately equal to the decay rate under making optimal measurements (red curve); a small decrease in the value of p_e increases the difference in the decay rates. However, this difference is less prominent at relatively longer measurement intervals, which is our primary regime of interest. Additionally, Figs. 6(c) and (d) show the difference in the decay rates as the value of σ varies. Once again, at relatively long intervals, the effective decay rate is quite unaffected by the imperfections in the measurement process.

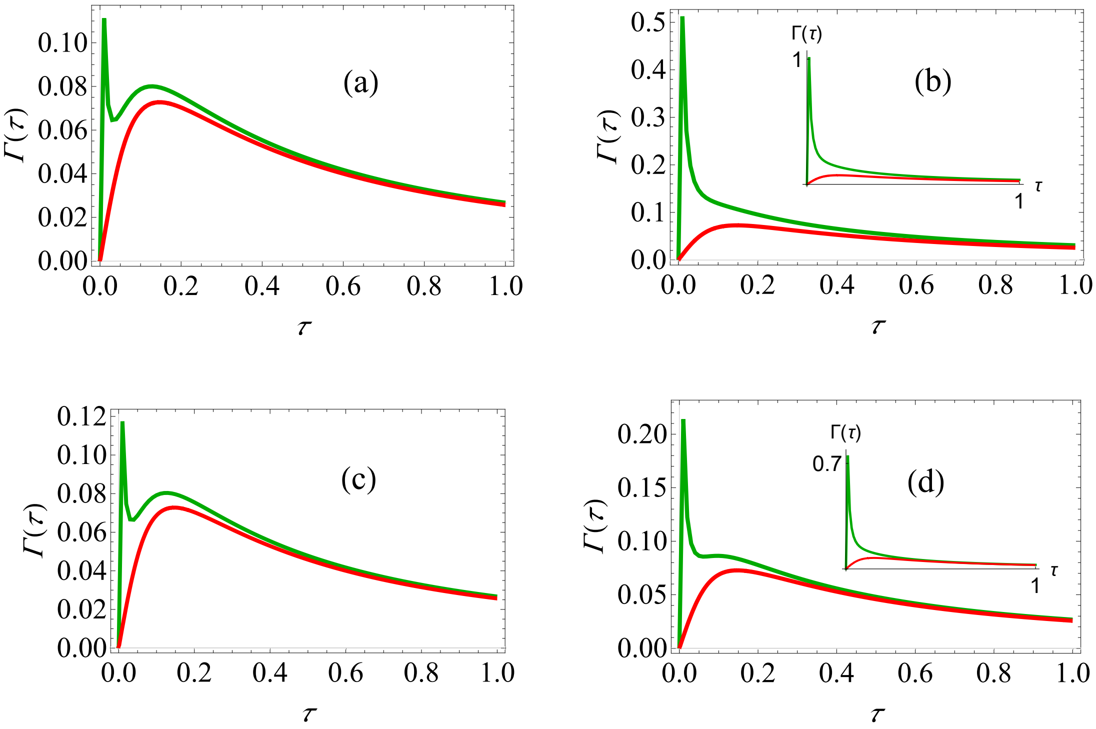

FIG. 6: **Behaviour of the effective decay rate for the pure dephasing model under non-ideal measurements. (a)** $\Gamma(\tau)$ versus τ. The red curve shows the decay rate if the optimal measurement is repeatedly made; the dark green curve shows the effective decay rate if the non-ideal measurement is made. We have considered the state specified by the Bloch vector $(1/\sqrt{10}, 0, \sqrt{9/10})$, with $G = 0.1, \beta = 0.5$ and $\omega_c = 10$. Additionally, we have chosen $P_e = 0.999, \sigma = \theta_0/100$. **(b)** Same as **(a)**, except now we have set $P_e = 0.995$. The inset shows the case where $P_e = 0.99$. **(c)** Same as **(a)**, except now we have set $\sigma = 0.05\theta_0$. **(d)** Same as **(c)**, except now we have set $\sigma = 0.2\theta_0$. The inset shows the case where $\sigma = 0.5\theta_0$.
